# Landmarks of the Knowledge and *Trypanosoma cruzi* Biology in the Wild Environment

**DOI:** 10.3389/fcimb.2020.00010

**Published:** 2020-02-06

**Authors:** Ana Maria Jansen, Samanta Cristina das Chagas Xavier, André Luiz R. Roque

**Affiliations:** Oswaldo Cruz Institute, Oswaldo Cruz Foundation, Rio de Janeiro, Brazil

**Keywords:** trypanosomatids, *Trypanosoma cruzi*, wild mammals, transmission cycle, reservoirs, spatial analysis

## Abstract

Trypanosomatids are ancient parasitic eukaryotes that still maintain prokaryotic characteristics. *Trypanosoma cruzi*, a primarily wild mammal parasite, infected humans already long before European colonization of the Americas. *T. cruzi* heterogeneity remains an unsolved question, and until now, it has still not been possible to associate *T. cruzi* genotypes with any biological or epidemiological feature. One of the first biochemical attempts to cluster the *T. cruzi* subpopulations recognized three main subpopulations (zymodemes) that have been associated with the transmission cycles in the wild (Z1; Z3) and in the domestic environment (Z2). The description of wild mammal species harboring Z2 two decades later challenged this assemblage attempt. Currently, the genotypes of *T. cruzi* are assembled in seven discrete typing units (DTUs). The biology of *T. cruzi* still shows novelties such as the description of epimastigotes multiplying and differentiating to metacyclic trypomastigotes in the lumen of the scent glands of *Didelphis* spp. and the capacity of the true meiosis in parallel to clonal reproduction. The study of the transmission cycle among wild animals has broken paradigms and raised new questions: (i) the interaction of the *T. cruzi* DTUs with each of its mammalian host species displays peculiarities; (ii) the impact of mixed genotypes and species on the transmissibility of one or another species or on pathogenesis is still unknown; (iii) independent *T. cruzi* transmission cycles may occur in the same forest fragment; (iv) the capacity to act as a reservoir depends on the peculiarities of the host species and the parasite genotype; and (v) faunistic composition is a defining trait of the *T. cruzi* transmission cycle profile. The development of models of environmental variables that determine the spatial distribution of the elements that make up *T. cruzi* transmission by spatial analysis, followed by map algebra and networking, are the next steps toward interpreting and dealing with the new profile of Chagas disease with its many peculiarities. There is no way to solve this neglected disease once and for all if not through a multidisciplinary look that takes into account all kinds of human and animal activities in parallel to environmental variations.

## Introduction

In addition to being a successful parasite, *T. cruzi* presents a very interesting story. It was first observed by Carlos Chagas in 1909 in the digestive tract of a triatomine (*Panstrongylus megistus*) examined after his attention was drawn to insects that sucked people's blood (Chagas, 1909). Since he was in Lassance, a poor little town in the interior of Minas Gerais State, and did not have laboratory facilities, Chagas asked his mentor and director, Oswaldo Cruz, to put infected triatomines in contact with marmosets (*Callithrix penicillata*). Three weeks later, Oswaldo Cruz observed trypomastigote forms in the blood of these animals. In contrast to what was thought by Chagas and Cruz (that the parasites were transmitted by the insects' bites), the marmosets probably became infected by the ingestion of the infected bugs, resulting in the first demonstration of the oral infection of wild mammals (Coura, 2009). Oswaldo Cruz urged Carlos Chagas to perform experimental infections in other animals soon done on mice, guinea pigs, rabbits, dogs and monkeys. In 1909, Carlos Chagas returned to Lassance, where he began to examine animals and humans. First, he identified trypomastigote forms in the blood of a cat; then, also in 1909, he detected the same trypomastigote forms in the blood of a child (Chagas, 1909). In 1912, Carlos Chagas described the presence of trypomastigotes in the blood of an armadillo and in a specimen of *Panstrongylus geniculatus* collected in the armadillo's burrow (Chagas, 1912). Chagas described the acute form of human disease in 1916 (Coura et al., 2014). Thus, a new species of *Trypanosoma*, its vector and mammalian hosts, and the human disease due to this trypanosome species were described by the same scientist, an exceptional fact in medicine. Despite having observed *T. cruzi* in one armadillo, Carlos Chagas did not link the wild and domestic cycles; moreover, the distance and lack of connectivity between the wild and domestic environments becomes very clear in the description of the armadillo as a “depositary of *T. cruzi* in the external world” (Chagas, 1912). In the same manuscript, for the first time, Chagas also suggested, based on the epidemiological data available, that *Triatoma infestans* was also implicated in *T. cruzi* transmission to humans. Later, this triatomine species was recognized as the most important domiciliated vector in several countries of South America.

*Trypanosoma cruzi* belongs to the family Trypanosomatidae, an ancient diverging eukaryotic monophyletic group of parasites that includes monoxenic and heteroxenic species. Trypanosomes still share many molecular characteristics with both prokaryotes and eukaryotes. Very recently, the meiotic machinery of higher eukaryotes has been described in *T. cruzi*. This mechanism was very early described in *T. b. brucei* (Gibson and Bailey, 1994; Gibson et al., 2008; Peacock et al., 2014), in *Leishmania* parasites (Akopyants et al., 2009), and in *T. congolense* (Van den Broeck et al., 2018), but not in *T. b. gambiense*, which was confirmed to be completely clonal (Schwabl et al., 2019).

Trypanosomatids are considered important because some of their representatives are etiological agents of important diseases for humans and animals of economic interest. This anthropocentric view drained the main study efforts for these species, providing poor attention to some other species that were associated with insects and, in a pejorative manner, called as “lower trypanosomatids.” With the awareness of the deep interdependence of environmental, human, animal and plant health, in addition to the emergence of molecular tools with higher analytical power, researchers have widened the interest of other taxa of trypanosomes and their hosts. This broader look also resulted in the awareness that the limits of parasite host specificity may be quite blurred. Thus, since 1980, cases of monoxenic trypanosomatids infecting humans in mixed infections with *Leishmania* spp. have been reported. These findings increased in number until the description of a fatal human case of a visceral leishmaniasis-like disease associated with a parasite related to *Crithidia* spp. (Maruyama et al., 2019). Wild free-ranging mammals infected solely by *Crithidia mellificae* have already been described (Rangel et al., 2019). These findings, in addition to the consciousness of the multifactorial character of the parasitism phenomenon and its consequences, have broken numerous paradigms. Among them, parasites are necessarily pathogenic agents, the concept of which is a reservoir and the evolution of the parasitism phenomenon, among others. A broader view of what is parasitism is important in the study of *Trypanosoma* spp., a ubiquitous taxon that displays complex life histories.

The importance of the consideration and study of the spatial characteristics in which the encounter between these actors, parasite and host, occurs, is becoming increasingly clear. In this sense, cartographic tools have been contributing to such an extent that they can no longer be dispensed. Filling in the gaps in the knowledge of these ancient eukaryotic organisms will surely improve our understanding of the complexity of the evolutionary process of living beings in general.

**Figure 1:**
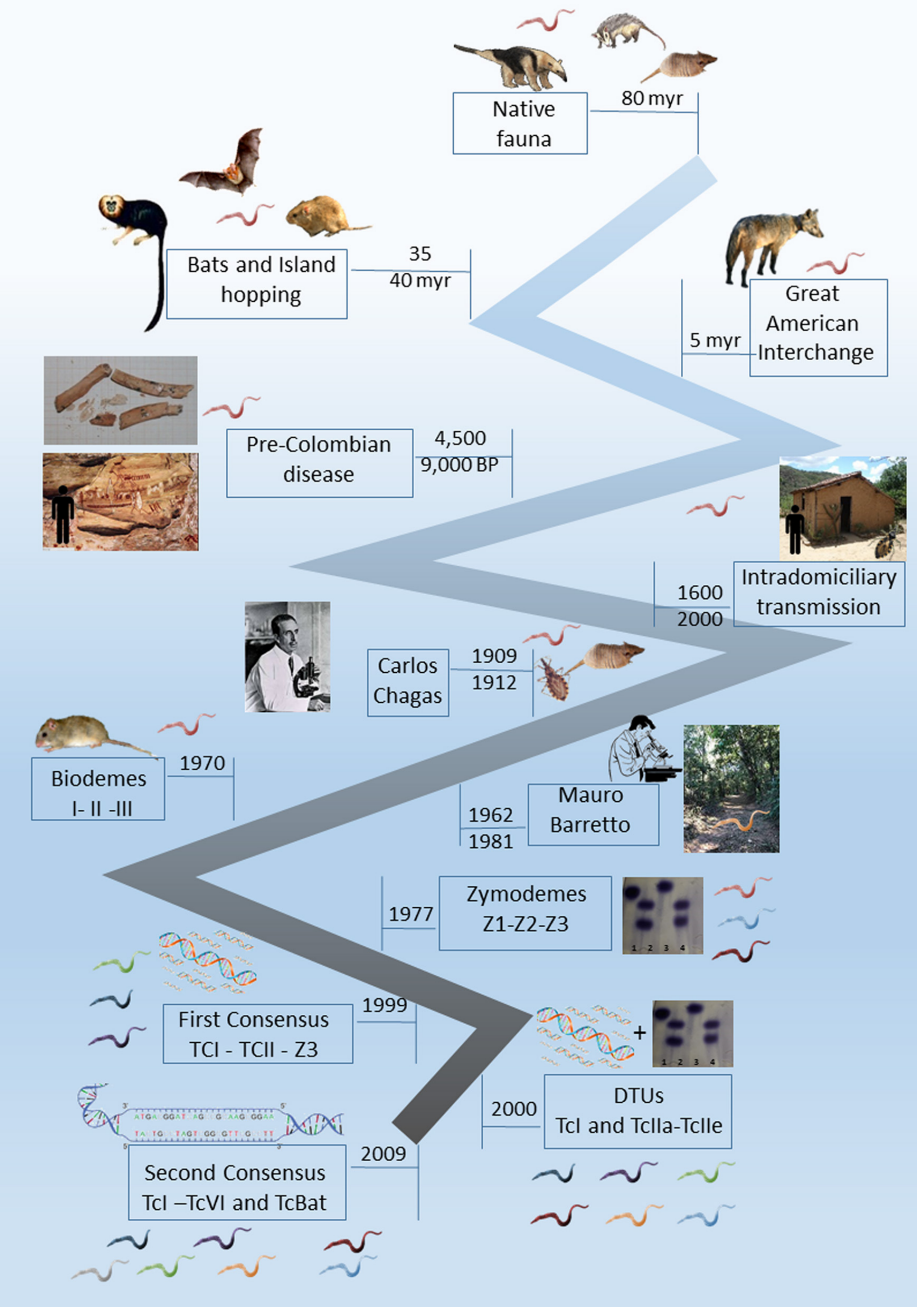
Timeline landmarks of the knowledge of *Trypanosoma cruzi* biology.

In this article, we will focus on *Trypanosoma cruzi*, not only as the etiological agent of Chagas disease but also as one of the most successful parasite species. We will mention here some key points from the longtime study and debate on the subject that has lasted for over a century (Figure 1). We will also present a current state of the art of the transmission cycle of *T. cruzi* in the wild as well as questions that are still controversial and unanswered.

## *Trypanosoma cruzi:* A Primarily Wild Mammal Parasite

*T. cruzi* infection was first described as an indoor transmitted trypanosomiasis involving humans living in poor housing and nocturnal domiciliated hematophagous bugs (*Conorhinus megistus*, now named *Panstrongylus megistus*) that lived in mudhouse walls (Chagas, 1909). In the following decades, some studies conducted especially in Brazil, Colombia, Venezuela and Costa Rica searched for *T. cruzi* in wild mammals, aiming to understand their role in the epidemiology of human disease. However, it was only during the seminal studies conducted by Mauro Pereira Barretto (from 1962 to 1981) in Brazil that this protozoan parasite started to be considered a primarily wild enzootic organism, maintained by wild mammals and triatomines (Barretto, 1967a). Nevertheless, the same author described trypanosomiasis due to *T. cruzi* as an amphixenosis that fitted very well in Pavlovsky's nidality postulate (Pavlovsky, 1967). Influenced by the studies of MP Barretto, the scientific community started to consider that the disruption of the ecological equilibrium in a wild environment could result in outbreaks of human disease. The main aspects observed by Barretto in that time (and now observed in the recent Chagas disease outbreaks) were (i) forest fragmentation, reducing areas for mammals and vectors; (ii) the displacement of host and vectors to new areas; (iii) proximity to human dwellings and their annexes; (iv) and the contact between infected vectors and humans (or human's prepared food or beverage) (Barretto, 1967b; Roque et al., 2008; Xavier et al., 2012). When Barretto finished his studies, he described more than one hundred mammal species naturally infected by *T. cruzi*, and the oral route was proposed to be the most efficient transmission via the wild. At that moment, this trypanosomiasis due to *T. cruzi* was no longer seen as a “human parasitosis” (Barretto and Ribeiro, 1979).

A very important landmark in the construction of the knowledge of this parasitosis was the discovery that humans became infected by *T. cruzi* probably since their arrival in the Americas and not just after South America's European colonization. *T. cruzi* infection was demonstrated in mummies from Chinchorro populations dated on 9,000 years BP (Aufderheide et al., 2004). Cardiac lesions compatible with the clinical outcome of chronic Chagas disease patients were also observed in mummies from the Chilean Atacama Desert (Rothhammer et al., 1985). In Brazil, the most ancient mummies found infected by this parasite are dated from 7,000 to 4,500 years BP and were derived from Central Brazil (Lima et al., 2008). In the Brazilian Northeast Region, ancient humans responsible for the rock paintings in one of the regions that display the most ancient registers of human presence (Serra da Capivara, in the Brazilian Northeast Region) were most likely exposed to infected *Triatoma brasiliensis* during the long time required to perform their paintings (Araújo et al., 2003). Accordingly, the domestication of *Cavia* sp. in the Andean Valley and the presence of wild mammals attracted to stocked grains adjacent to human dwellings were important factors for the establishment of the domiciliary populations of *T. infestans* (and consequently, the intradomiciliary transmission scenario described by Chagas) (Dias and Coura, 1997).

## *T. cruzi* Genotypes and its Ecology: A Phenomenon Not Yet Fully Understood

The heterogeneity of *T. cruzi* had already been observed by Carlos Chagas, who detected slim and wide blood trypomastigotes and, most likely influenced by his experience with malaria, attributed the different forms to male and female merozoites (Chagas, 1909). Later, several authors described different patterns of cell growth and differentiation in axenic cultures, demonstrating that the heterogeneity observed was not only morphological but could result in distinct success in replicating and differentiating into infective forms.

The first attempt to cluster the observed heterogeneity among *T. cruzi* subpopulations was proposed by Sonia Andrade in the 1970s and was based on the different infection patterns observed in Swiss mice experimentally infected with different *T. cruzi* strains. Three Biodemes were proposed: Biodeme I for strains that resulted in high virulence and mortality in 10–12 dpi; Biodeme II in the cases of mild virulence and mortality after only 20–25 dpi; and Biodeme III, which resulted in a slow increase in parasitemia and no mortality in infected mice (Andrade et al., 1970; Andrade and Magalhães, 1997). This proposition, however, was time-consuming, dependent on laboratory animal availability, and isolates certainly underwent selection pressure due to the experimental infection. Brener (1977) suggested the classification of two polar types based on morphology and tissue tropism, describing an aggressive pole represented by the Y strain and a benign pole exemplified by the CL strain. The biological, immunological, drug resistance and clinical differences that were being unveiled were so impressive that it was even proposed to consider that *T. cruzi* was in fact a species complex and not a single species and that the taxon should be referred to as the “*cruzi* complex” (Coura et al., 1966).

The characterization of *T. cruzi* subpopulations by a biochemical tool was successfully proposed by Michael Miles and coauthors in 1977 based on enzyme electrophoresis (Miles et al., 1977). This proposition was certainly one of the most important landmarks in the study of *T. cruzi* biology, and the three main zymodeme groups proposed (Z1, Z2, and Z3) were the basis for the first nomenclature consensus proposed in 1999 (Anonymous, 1999). Another important zymodeme described in the Southern Cone of South America was the so-called Z2 Bolivian or Zymodeme 39 (named TcV in the current *T. cruzi* nomenclature), which is a hybrid parasite that is the result of a natural product of meiosis recombination between the parentals TcII and TcIII. This *T. cruzi* genotype was described by its heterozygous profile of the isoenzyme glucose phosphate isomerase, which gave important information long ago (Apt et al., 1987; Brenière et al., 1989). The majority of the molecular studies on *T. cruzi* that started to be conducted approximately a decade later confirmed the differentiation into the two main (and parental) groups, *T. cruzi* TcI (Z1) and TcII (Z2) (Clark and Pung, 1994; Tibayrenc, 1995; Souto et al., 1996).

Since their description, the different zymodemes were associated with different transmission cycles: Z1 and Z3 were associated with the transmission cycles in the wild and Z2 in the domestic environment. The domestic cycle of *T. cruzi* transmission was proposed to be somewhat independent from the sylvatic one, although sometimes overlapping (Miles et al., 1980; Apt et al., 1987; Zingales et al., 1998). This proposition was supported, in part, by two important features: (i) *T. cruzi* TcI (Z1) is the most ubiquitous subpopulation and, because of that, is the most frequently detected subpopulation in the wild (Fernandes et al., 1999; Noireau et al., 2009; Jansen et al., 2015); and (ii) *T. cruzi* TcII (Z2) was the subpopulation that was consistently isolated from human cases in the formerly endemic areas of Central Brazil, especially maintained by *T. infestans* in indoor transmission. This subpopulation was therefore associated with the domestic cycle of *T. cruzi* transmission and with the severe clinical conditions that occurred in 30% of the infected people (Chapman et al., 1984; Fernandes et al., 1999). It was more than two decades later, through the description of a well-established transmission cycle involving free-ranging golden lion tamarins (GLTs; *Leontopithecus rosalia*) and *T. cruzi* Z2 subpopulations (later confirmed as DTU TcII), that this association was challenged (Lisboa et al., 2000, 2006). Moreover, the infection by *T. cruzi* TcII in GLTs was followed up for more than a decade and was demonstrated to be the most stable and expressive transmission cycle of this DTU in the wild (Lisboa et al., 2015). Since these initial findings, *T. cruzi* DTU TcII has been described in several other wild mammal species throughout Latin America, including rodents, marsupials, bats and carnivores (Jansen et al., 2015). To date, it has not been possible to unequivocally associate *T. cruzi* genotypes with any biological response variable, including the biome and environment (as first proposed) or host species (as proposed secondly).

The advances of the molecular techniques for *T. cruzi* characterization since the late 1980s, and the most diverse targets proposed, showed that *T. cruzi* heterogeneity was much higher than the biochemical studies could reveal and made it possible to study the parasite at a much higher level of detail. Soon after the first nomenclature consensus was published, six discrete phylogenetic lineages (later named discrete typing units—DTUs) were proposed (Brisse et al., 2000). The former Z1 and Z2 comprised one DTU each, Z3 was divided into 2 distinct DTUs, and the hybrid isolates were grouped into two other DTUs. This division was based on the second (and currently valid) nomenclature consensus that divided the *T. cruzi* subpopulations into 6 DTUs, named TcI to TcVI (Zingales et al., 2009). A putative seventh DTU, Tcbat, was described as a DTU associated with bats, although human infections by this DTU were already reported (Marcili et al., 2009; Guhl et al., 2014; Ramírez et al., 2014).

It is not surprising that TcI was initially associated with the sylvatic cycles because TcI is the most widespread DTU, being detected in all areas of *T. cruzi* transmission (Jansen et al., 2015). This DTU was first associated with opossums; however, while individuals from the *Didelphis* genus were most commonly infected by TcI (Fernandes et al., 1999; Jansen et al., 2015; Roman et al., 2018b), individuals from the *Philander* genus were found to be infected by both TcI and TcII at similar rates (Fernandes et al., 1999; Pinho et al., 2000; Jansen et al., 2015). *T. cruzi* TcII infection may be detected in concomitant infection with TcI in *Didelphis* individuals (Jansen et al., 2018). The proposed association among TcI, *Didelphis* sp. and the arboreal stratum (Yeo et al., 2005) did not consider that *Didelphis* spp. are scansorial and not arboreal mammals, i.e., *Didelphis* sp. may also use the terrestrial and arboreal strata. This DTU, along with DTUs III and IV, is involved in human cases in the Amazonian basin, currently the region that reported more than 90% of new cases in Brazil and severe acute phases (Monteiro W. M. et al., 2010; Monteiro et al., 2012).

The DTU TcII included *T. cruzi* subpopulations derived from patients from formerly endemic areas, where clinical symptoms of chronic Chagas disease were common and severe, and because of that, this DTU was associated with a domestic cycle of parasite transmission. Reports on TcII-infected wild mammals are less numerous in comparison to those on TcI, but TcII also presents a noteworthy host range and is widely distributed in nature (Jansen et al., 2015). TcII is the second most frequently found genotype infecting wild mammals in Brazil, including mammals of the Amazon basin region (Lima et al., 2014).

Undoubtedly a turning point, an important milestone in the discussion of genetic diversity in *T. cruzi* was the recent description of panmixia in some *T. cruzi* subpopulations (Schwabl et al., 2019). The description of this phenomenon in some isolates of Ecuador sheds light on a decades-long debate related to the largely clonal character of the taxon. More intriguing is the description of clonal groups that may occur in sympatry with clonal groups that the authors propose to have experienced in past hybridization events. These fascinating findings largely explain the extreme diversity of *T. cruzi* and its extreme adaptability to species and tissues of its numerous vertebrate and invertebrate hosts Additionally, the studies of Schwabl and coauthors postpone further the possibility of understanding the ecological significance of the genetic diversity of *T. cruzi* (Schwabl et al., 2019).

## Novelties on *T. cruzi* Biology Shown By Opossums

Another milestone in the history of the study of *T. cruzi* biology came in 1984 with the discovery of extracellular forms of *T. cruzi* (epimastigote and trypomastigote) in *Didelphis aurita* opossum smelling glands (Deane et al., 1984a). This finding showed that opossums can act as reservoirs and vectors of *T. cruzi*.

Opossums, like many other mammals, have a pair of anal glands. These glands have a wrap that is partly made up of a striated muscle layer and a small portion of pearly looking connective tissue. When threatened or stressed, these animals expel the extremely bad smelling content of these glands as a defense mechanism. Within the scent glands, the flagellates are preferentially disposed around the glandular epithelium, an area rich in hyaluronic acid. That is, the scent glands do not constitute a reservoir within the reservoir. Interestingly, when injected directly into the scent glands, *Leptomonas* sp. and *Chrithidia* sp. are able to establish stable infections, surviving, multiplying and even eliciting a humoral immune response. Taken together, these findings led Deane and coauthors to propose scent glands as the steppingstone for the adaptation of *T. cruzi* to this mammalian host that they considered the more ancient host of *T. cruzi* (Deane et al., 1984a).

Maria Deane and her colleagues conducted a pioneering long-term study on the interaction of *T. cruzi* with the opossum *D. aurita*. These studies included experimentally infected animals born in captivity and naturally infected animals. Didelphid opossums are likely to be ancient *T. cruzi* hosts, and that likelihood was the authors' motivation for the studies. These studies showed that *D. aurita* displayed a very distinct infection pattern when infected by the Y strain (TcII) in comparison to the infections caused by the F strain (TcI): opossums were shown to rapidly eliminate the former from the peripheral circulation while maintaining blood-detectable parasitemias when infected by the latter. In other words, it became clear that opossums differ in their infectious potential for TcI and TcII. Moreover, these studies have shown that from an early age, approximately 50 days but still in the marsupial pouch, *D. aurita* is able to control *T. cruzi* infection (Jansen et al., 1994). This does not apply to newborns because didelphids are born still at the embryonic stage and therefore very immature. Newborn didelphids do not reject grafts, do not have individualized back paws, and their eyes and ears are sealed; therefore, they are entirely dependent on the incubation conditions of the marsupium for their survival. Deane's findings shed light on an important aspect of *T. cruzi* biology—the peculiar character of the interaction of *T. cruzi* with its mammalian hosts. Accordingly, it was observed that *D. aurita* maintains high parasitemias by DTU TcI but not by DTU TcII in contrast to another didelphid marsupial species, *Philander opossum*, that responds with high parasitemias when inoculated with DTU TcII (Legey et al., 1999). Further studies on wild mammals have shown the complexity of *T. cruzi* transmission cycles in the natural environment. It has been seen that different *T. cruzi* DTUs can infect the same mammal and be transmitted by the same triatomine specimen. Studies of experimental *T. cruzi* infection in opossum initiated by Maria Deane were a paradigm break because they showed that strain Y, considered “aggressive,” was far from deserving of this adjective in opossum infections. High intraperitoneal Y strain inocula are in fact extremely virulent and pathogenic to mice (Andrade et al., 1970; Andrade and Magalhães, 1997). The findings of Maria Deane reinforced that virulence and pathogenicity are not exclusive attributes of the parasite and that virulence does not necessarily result in pathogenicity. Indeed, opossums maintain long-lasting high parasitemias by F (TcI strain) that are detectable by blood cultures for a long time without significant damage (Jansen et al., 1991). Additionally, golden lion tamarins (GLTs) are able to maintain natural infections by *T. cruzi* TcII without clinical biochemical or cardiological impairment (Monteiro R. V. et al., 2010; Lisboa et al., 2015).

An interesting aspect of the interaction of *T. cruzi* with opossums is the absence of congenital or neonatal transmission in *D. aurita*. The hypothesis that the absence of a placenta and the long period of breastfeeding of didelphids could result in transmission to neonates was not confirmed. Furthermore, the newly weaned puppies showed some protection against experimental infection, probably due to the antibodies passed during the long period of breastfeeding. The reproductive investment of opossums is postpartum: after a short gestational period of 13 days, neonates maintain themselves attached to the nipples. It is only at 45 days after birth that the newborns begin to try other foodstuffs, but they still return to the marsupial pouch. The total independence of the young happens approximately 100 days after birth (Jansen et al., 1994). These studies were only possible after the standardization of a serological test to diagnose *T. cruzi* infection in opossums. In nature, most infected animals do not have parasitemia detectable by fresh blood smear examination or blood culture; therefore, serological tests are important. However, this is an important bottleneck—there are simply no commercial serological tests on the market. We adapted an indirect immunofluorescence reaction by including a rabbit elicited intermediate anti-opossum IgG antibody, revealing the reaction with a commercial anti-rabbit IgG fluorescein antibody (Jansen et al., 1985). Recently, one of us (SCX) started to purify and label with fluorescein an anti-opossum IgG.

*T. cruzi* infection in wild mammals displays an aggregated distribution, occurs in the six biomes of Brazil (a country of continental dimensions) and involves all mammalian orders. It is worth mentioning that parasitemias are not high. Rarely are positive fresh blood tests reported, and positive blood cultures that also demonstrate infective potential were observed in 8% of 6,587 wild mammals examined (Jansen et al., 2018). Procyonidae (*Nasua nasua*), Primates (*Leontopithecus rosalia* and *Sapajus libidinosus*), and Marsupialia (especially *Philander* spp. and *Didelphis* spp.) more frequently demonstrate positive hemocultures, i.e., infectious potential, than other taxa (Jansen et al., 2018).

The transmission of *T. cruzi* may occur between all animals of a given forest stratum or be limited to one stratum only, as observed by Fernandes et al. (1999). This implies important consequences, the first being the importance of the accuracy of the sample calculation of both species and the number of animals to be examined. Failure to take into account that, even within the same forest fragment, independent transmission cycles may be taking place will certainly result in important biases that may compromise environmental management decision-making or the determination of epidemiological risk factors for public health. To determine which animal species play the role of the reservoir, we need to examine a representative number of representative species. For example, in the southeastern Atlantic rainforest (Rio de Janeiro), golden lion tamarins are the reservoirs of *T. cruzi* and not *D. aurita*, a species that is classically defined as a *T. cruzi* reservoir (Lisboa et al., 2015).

Considering the peculiarities in the interaction of *T. cruzi* with the mammalian host species and that *T. cruzi* is able to infect hundreds of mammalian species, one can compare the enzootic scenario of transmission in the wild to the ever-changing figures of a kaleidoscope resulting from the countless arrangements of its colored components. As the faunal composition of each locality defines the enzootic scenario, assessing the risk of human infection necessarily includes considering, beyond the triatomine fauna, the mammalian hosts and reservoirs.

## Are Laboratory *T. cruzi* Strains Representative of the Natural Populations?

*T. cruzi* is a highly diverse taxon expressed by differences in infectious competences, generation time and life strategies, as exemplified by the existence of cloned subpopulations and sexually reproducing subpopulations within the taxon (Berry et al., 2019). These characteristics make the study of this parasite very difficult because it raises the question of the representativeness of laboratory samples and raises the question of the reproducibility in the laboratory of what happens in nature. Two difficult obstacles to overcome show that there are no representative laboratory samples: the subsampling and the selective pressures inherent to laboratory maintenance methods exerted on natural populations of *T. cruzi*.

Subsampling seems very probable considering that *T. cruzi* infects hundreds of host species that are transmitted in complex life cycles, from the southern United States to southern Argentina. Representative samples of both animal species as well as of regions and even biomes are unlikely to have been collected. Clonal heterogeneity in *T. cruzi* was long ago demonstrated by biological, biochemical, immunochemical, parasitological, and histopathological parameters by several authors (Lanar et al., 1981; Dvorak, 1984; Tibayrenc et al., 1986). More recently, molecular analysis methods showed that there is high diversity even within DTUs (Roman et al., 2018a,b).

The already classic reference strains, such as the Y, Colombiana, Esmereldo, CL, and other strains, are highly passaged strains, kept for many years in laboratories, and are therefore subjected to the selective pressures inherent to these methods and hardly represent the diversity of *T. cruzi*. The selection of *T. cruzi* strains according to the experimental protocol was observed decades ago in experimentally infected mice (Deane et al., 1984b). The observed 10-fold difference in the rate of growth of epimastigotes (Dvorak, 1984) shows that many biological clones are lost by “*in vivo*” or “*in vitro*” passages. Clonal selection certainly also occurs in the natural environment and is directed by the host species (mammal or triatomine) infection route and other factors. Considering that each species (and even individual) interacts differently with the different subpopulations of *T. cruzi*, it is possible to conclude that the possibilities of combinations are endless.

Given that the true diversity of *T. cruzi* is very likely underestimated, attempts to associate parasite genotypes with host species or environment-related variables should be proposed with caution. Finally, when faced with such a complex model, it is prudent not to dogmatize and to always be open to reviewing concepts.

## Human Infection and Disease After *T. infestans* Control

Although Barretto has drawn attention to the correlation between environmental disruption and the onset of human disease, this knowledge has not been incorporated into the routine of preventive actions. Nor could it be incorporated because the transmission of *T. cruzi* to humans was basically intradomiciliar by *T. infestans*, an exotic species in Brazil that interestingly never adapted to the wild environment of any Brazilian biome. Therefore, the successful campaign to eliminate indoor *T. cruzi* transmission by *T. infestans* was based on single measures that could be taken in all areas of transmission, including the spraying of houses and attachments, educational campaigns and *T. infestans* collections. Thus, it was possible to use the same vector combat methodology throughout.

Despite the focus of the present study not being human disease or infection, the control of intradomicile *T. cruzi* transmission by *T. infestans* in Brazil is an issue worth mentioning. This was indeed a very important landmark. In fact, the Chagas Disease Control Program established in Brazil in 1975 achieved, in this continental-sized country, a highly successful campaign, starting from millions of infected people to zero in 25 years (Schofield and Maudlin, 2001). In terms of public health, this was an enormous milestone. Even though it was not technically complicated, it was necessary to train and supervise a large number of men who had to undergo hard work in some cases in very severe climatic conditions, working under standard protocols across the vast area, all without today's communication facilities. An additional difficulty was the local peculiarities of several regions of the country. It was proposed that the control of *T. infestans* in the Northeast would be easier to fight because of the short time of existence of this triatomine in the region and a putative lower adaptation level to the conditions of that biome (Ramos and Carvalho, 2001). Will there be intrahome transmission of *T. cruzi* again? It is hard to know. In addition to the improvements that have occurred in several housings, it was proposed that habitat change and adaptation to a new environment constitute a long evolutionary process in triatomines (Schofield et al., 1999).

Currently, Chagas disease is of concern again but with an entirely new, challenging and very complex epidemiological profile. Trypanosomiasis by *T. cruzi* is a neglected disease despite being a reemerging parasitosis and is no longer restricted to the new world. This spillover to nonendemic areas, including other continents out of the Americas, is mainly due to uncontrolled migratory movements, blood transfusion and organ transplantation (Franco-Paredes et al., 2009). Additionally, the enzootic nature of Chagas disease has been extending its limits, as has been observed in the southern United States, where an increase in the number of infected dogs as well as an increase in the areas of occurrence have been described (Curtis-Robles et al., 2017). Dogs are the last barrier between the wild cycle of transmission and the human environment. A recent review on the subject estimated that there are 300,000 *T. cruzi*-infected humans in the United States (Andrade et al., 2014). This number is likely underestimated.

## Outbreaks and Acute Cases of Chagas Disease

In Latin America, outbreaks and cases of oral Chagas disease have been increasing worryingly, especially because, in general, they result in severe clinical manifestations contrary to what is known of this infection in animals. Opossums fed on infected triatomine or mice serologically convert much later than when infected subcutaneously and do not have patent parasitemia like humans. What makes the early diagnosis of Chagas disease fundamental is the increased chance of cure of the infected individuals who has early access to treatment and the increased chance of those infected and precociously treated individuals remaining asymptomatic and not progressing to the severe forms of disease.

What makes early detection of *T. cruzi* infection so difficult is, in addition to the lack of local prepared staff, the lack of pathognomonic signs and the diversity of epidemiological scenarios in which oral cases and outbreaks occur. In fact, although *T. cruzi* infection is always associated with the consumption of food contaminated by infected triatomines, there are numerous variables that are still unknown. Thus, the outbreak of Chagas disease that occurred in 2005 in southern Brazil, in Santa Catarina, related to the consumption of contaminated sugarcane juice, raised several tantalizing hypotheses that were being discarded as the real cause was brought to light as, for example, sugarcane plantations would favor the multiplication of triatomines. It was found that the contamination occurred at the sugarcane mill located near a window in which infected triatomines fell into from a neighboring palm tree probably attracted by light (Roque et al., 2008; Steindel et al., 2008). The origin of the contaminated food that resulted in the outbreak reported in Redenção/CE in the northern part of Brazil was never clarified because the wild transmission cycle of *T. cruzi* was observed in different parts of the municipality (Roque et al., 2008). Despite the presence of *T. cruzi*-infected wild mammals in the surroundings (as is the common scenario throughout the country), both municipalities displayed good sanitary conditions and the predominance of an urban profile. Humans in those areas did not expose themselves or their food to triatomine bugs. There was never a case or outbreak in these localities again.

A contrasting transmission scenario was observed in the Amazon region, where contaminated açai fruit (*Euterpe oleracea*) juice was associated with the great majority of current cases in Brazil (Santos et al., 2018). In these outbreaks, the precariousness of local sanitary and educational conditions associated with the presence of infected bugs, eventually infesting the panniers used to transport the fruits, resulted in human cases, always in the more dry and hot period of the year, coinciding with both the açai harvest and the increased flying activities of the bugs (Xavier et al., 2014). Several cases were reported in the riverside population, usually as a familial outbreak. The difficulties in access to health professionals, the lack of information about the transmission and symptoms and, mainly, the complete absence of sanitary care likely resulted in the underestimation of such outbreaks.

A common conceptual error is that triatomine bugs are found colonizing açai palm trees. In fact, the distribution of bugs in Amazonian palm trees is neither homogeneous nor random but is much more intense in palm trees with higher accumulation of organic material (Abad-Franch et al., 2010). This is the opposite characteristic of the açai palm trees. The attracting factor for bugs starts after the açai fruit collection and is associated with the harvest, transport and characteristics of the fruit itself. After harvest, a natural fermentation process of the fruit produces carbonic gas, heat and humidity that can attract insects, especially in the sunset, when hundreds of açai panniers are maintained on the riverbanks near artificial light while the boats arrive to transport the fruits to be negotiated. These are the conditions in which the fruits are sold in Belém, one of the largest municipalities of the Brazilian Amazon region and the one that reports a higher number of new cases of ACD annually. Infected triatomines transported with açai fruits and derived from nearby islands are responsible for the contamination of juices that are sold in the urban areas of Belém, in an epidemiological feature named “*Distantiae Transmission*” (Xavier et al., 2014). This feature may also occur when one beverage is produced (and contaminated) in one area but is consumed and responsible for infecting people in other areas. This situation occurred in the largest Chagas disease oral outbreak ever reported in a school in the urban area of Caracas, Venezuela. Guava juice produced in a rural area was contaminated with feces of infected *Panstrongylus geniculatus* and served in the school, resulting in more than one hundred cases of Chagas disease (Alarcón de Noya et al., 2010).

In addition to beverages, *T. cruzi* may be transmitted through different comestibles, even by solid food. In 2009, one familiar Chagas disease outbreak was reported in Tocantins State, Brazil, associated with the consumption of babaçu palm heart. In this case, the instrument used to cut vegetation probably came in contact with an infected bug (probably cutting its body), and this same instrument was used to cut the palm heart before distributing it to the families. Additionally, in the same state, an outbreak in Ananás municipality in 2012 received attention due to the high number of infected people (12 cases). This latter case was associated with the consumption of bacaba fruit (*Oenocarpus bacaba*) juice, produced in a similar manner as açai, but with cultural differences associated with its consumption that can directly impact the number of infected people. In this sense, açai fruit juice is consumed daily in some Amazon areas, usually immediately after its preparation and only by the family due to its low income. Contaminated açai juices result in familial Chagas disease outbreaks, usually involving 4 or 5 people. On the other hand, bacaba juice is sporadically consumed and, due to its high output (up to 10 liters), it is usually consumed with friends and for more than 1 day. Contaminated juices, in this feature, can result in an increase in the number of infected people, as was the case of the outbreak reported in Ananás. The most recent outbreak in Brazil occurred in September 2019, resulting in the infection of 16 individuals, and was associated with the consumption of the fruit of the patawa tree (*Oenocarpus bataua*), a palm tree that reaches 25 m height. This highly appreciated palm tree is found in the Amazon basin, and its fruits are used for several purposes, including juice preparation. Altogether, these cases demonstrate that, more than a specific food, human outbreak cases are the result of poor sanitary education and poor practices of food manipulation.

Oral infection may occur directly by contact with infected triatomine feces, independent of comestibles, as was observed by fatal case of a 2-year-old boy from Guarapari, in the Brazilian Southeast Region. The infection was associated with *Triatoma vitticeps*, a triatomine considered to be a secondary vector due to its delay of defecation after a blood meal but that demonstrated to be quite efficient in concomitantly hosting several *T. cruzi* DTUs and *T. dionisii*. The boy was infected by handling this *T. vitticeps* specimen (Dario et al., 2016).

One of the largest Brazilian outbreaks of Chagas disease occurred in Ibimirim, in the Brazilian Northeast Region, and was tentatively associated with food. Moreover, the real source of infection that resulted in 30 treated individuals was not yet identified 6 months later. Certainly, it was not due to the most commonly described beverages in other outbreaks (sugarcane, açai, or bacaba). These distinct epidemiological scenarios throughout Brazil show the necessity of distinct control measures.

The definitive control of Chagas disease is possible and, more than that, is urgent. It should not be possible that this parasitosis is still a scourge more than one hundred years after its discovery and so much accumulated knowledge. However, control will be possible only under an integrated multidisciplinary focus and not by the traditional control measures. Spraying in this new epidemiological scenario is the worst control method. The development of an understanding of all cultural, social and economic realities of the outbreak areas and, obviously, all epidemiological features, including fauna composition hosts and vectors, will be the only way to define and adopt the correct measures that must include economic, health and educational improvement.

## *T. cruzi* and its Successful Transmission Strategy in Nature

Pioneers in epidemiology postulated that all infected organisms are equally capable of infecting other organisms; that is, they have similar infectious competence. Further studies did not confirm this postulate but proposed the 20/80 rule; that is, a few individuals (humans or animals) are responsible for controlling transmission (Woolhouse et al., 1997). Obviously, the emergence of a superspreader is also regulated by other variables, such as immunosuppression and concomitant infections. This phenomenon has already been described in numerous parasitic diseases, including *Leishmania infantum* (Duthie et al., 2018).

**Figure 2:**
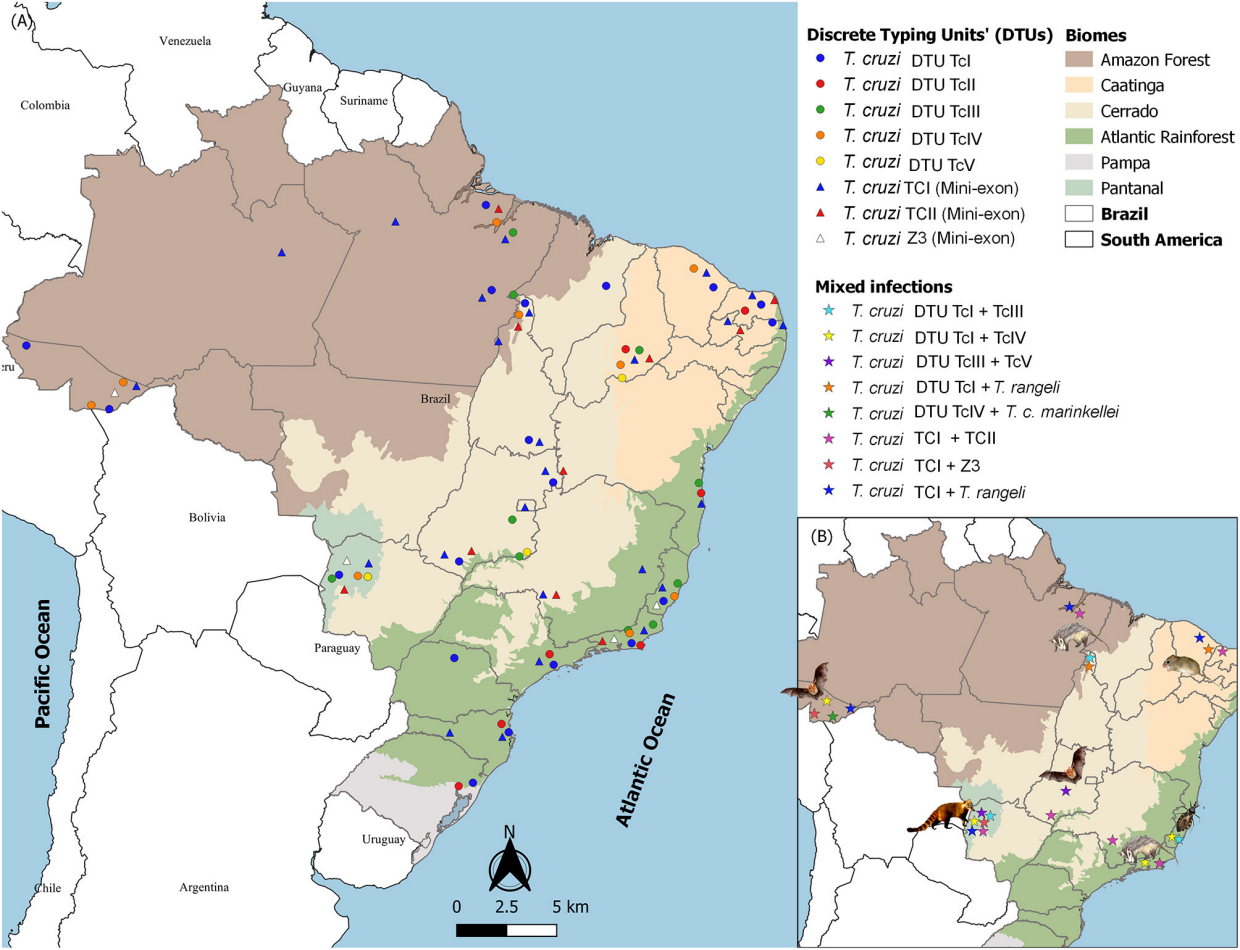
Distribution of *Trypanosoma cruzi* DTUs in Brazilian biomes according to the isolates deposited in the Coleção de *Trypanosoma* de Mamíferos Silvestres, Domésticos e Vetores (http://coltryp.fiocruz.br) **(A)** and the observed mixed infections and their hosts in Brazil **(B)**.

*Trypanosoma cruzi* is a mammalian hematozoan that depends on an insect vector for its passage to another mammalian host. The chances of infecting a hematophagous insect are increased, among others, if the parasitic population in peripheral blood is high. Interestingly, parasites in fresh blood smears or other blood tests of infected mammals are extremely rare in Brazil, independent of the *T. cruzi* DTU. Positive blood cultures or positive xenodiagnoses are parasitological enrichment methods that indicate high parasitemia and, therefore, the infectious potential of the animal. Working with hemocultures under field conditions is safer than working with xenodiagnoses, which includes a longer restraining time of the animal and is not free of accidents that result in the evasion of insects. The examination of 6,587 free-ranging wild mammals showed that 8% of all tested animals displayed positive blood cultures. Figure 2A shows the distribution of *T. cruzi* DTUs in Brazil, a country of continental dimensions and a large variety of environments and biocenoses. Note that TcI and TcII are the most frequently encountered DTUs in all biomes, followed by TcIII and TcIV. TcV is the most rarely found DTU. Four aspects deserve to be highlighted: (i) the transmission strategies of the less frequent DTUs; (ii) the fact that these DTUs are detected at extremely distant points; (iii) the lack of association of DTUs with biomes; and (iv) the high diversity of DTUs in a relatively small area of the southeastern Atlantic Forest. Increasing studies of habitats and areas will probably add information that may change this map. All of these questions show that the biological history of *T. cruzi* is far from complete. Numerous chapters are still to be written about the biological history of *T. cruzi* that will likely include even more spectacular landmarks than those highlighted here.

Still considering the positive hemocultures, opossums, mainly *Philander* spp. and *Didelphis* spp., the coati *Nasua nasua*, the capuchin monkey *Sapajus libidinosus* and the golden lion tamarin *Leontopithecus rosalia*, were the mammalians that demonstrated higher rates of positivity, demonstrating infectious potential (Jansen et al., 2018). This does not seem to be a superspreader maintenance strategy. The possible survival strategy of *T. cruzi* in the wild seems to depend on the sum of the high parasitemia periods of the assemblage of individuals and animal species that make up a particular community. Apparently, most mammalian species have high parasitemia periods for short times, i.e., infectious potential for a limited time. Only some species have a long period of high parasitemia detectable by blood culture, which means a long period of infectious potential, as mentioned above. Most likely, infected animals with negative blood culture had already passed their early stages of infection and infectious potential. To validate this hypothesis, it would be necessary to have representative samples of *T. cruzi* and mammalian species.

Another question refers to the permanence of the less frequent *T. cruzi* genotypes in nature. The two possible hypotheses are (i) that at some point in the infection, these genotypes had a high circulating blood population; or (ii) other unknown transmission mechanisms warrant transmission in low parasitemias, as is the case in Brazil of the DTUs TcV and TcVI that are rarely found in nature. A plausible explanation can be drawn from a Santa Catarina outbreak in 2005. At that location, the wild mammal fauna was restricted to marsupials of the genus *Didelphis* whose blood cultures indicated in TcI in contrast to the human cases that were all by TcII. One specimen of *Triatoma tibiamaculata* had a mixed TcI and TcII infection (Roque et al., 2008). The mystery was only resolved by PCR of *Didelphis* serum, which showed the presence of TcII in the opossum (Lima et al., 2014). As has been described, *Didelphis* rapidly controls TcII infections at subpatent levels (Jansen et al., 1991).

A clear demonstration of how biased associations can be established is provided by Guarapari's case of the child who became orally infected by *T. cruzi* by his hand that had manipulated an infected triatomine (Dario et al., 2016). This child presented infection in the cardiac tissue by DTUs TcI, TcII, TcIII, TcIV, and *T. dionisii*. Reports of mixed human infections with such diversity of *T. cruzi* taxonomic units are very rare, especially by *T. dionisii*, which is a bat-associated trypanosome species. This is most likely due to the rarity of histopathological examinations followed by the molecular characterization of tissues of individuals in the acute phase, i.e., very recently infected. This individual would likely not maintain such diversity indefinitely. Infections by some DTUs could be self-solving. At least *T. dionisii*, the bat-associated species, would probably have been eliminated or at least remained as a cryptic infection. Additionally, even total blood PCR is not necessarily representative of the richness of trypanomatid genotypes and species that infect a particular individual, not to mention the selective pressures exerted on the parasites by the immune response. The parasitological history of an individual is totally ignored when it is examined at later stages of infection. This results in at least two biases: (i) clinical manifestations are attributed only to that species or genotype that eventually is detected, and the possible effects of an initial multiple infection are totally ignored; and (ii) associations between the host and parasite (species or genotypes) are established based only on one single finding that is very probably not necessarily representative.

There are still many open questions on this topic, one of which specifically refers to the transmission of these genotypes and species that are so rarely found in nature. Are they becoming extinct, or are they beginning a process of expansion?

## Mixed Infections

Mixed infections by species or by subpopulations of the same species are common phenomena in nature. They are difficult to follow because wildlife capture and recapture studies require very expensive infrastructure as well as specific diagnostic kits. However, although recognized as a potential selective filter, 16% of molecular characterization on the cultures derived from wild mammal's blood or infected triatomine feces revealed mixed infections, with TcI and TcII being the most frequent combination (Jansen et al., 2015). Mixed infections by distinct *T. cruzi* DTUs or *Trypanosoma* species were observed in all Brazilian biomes, but the host most frequently associated in these mixed infections varies: bats in the Amazon Forest and Cerrado, opossums also in the Amazon Forest and Atlantic Rainforest, coatis in Pantanal, caviomorph rodents from the *Thrichomys* genus in Caatinga and triatomines, mainly *Triatoma vitticeps*, in the Atlantic Rainforest (Figure 2B).

Theoretical models predict an increase in virulence based on the competitive advantage of the most virulent subpopulations (Cressler et al., 2016). This would be the case with competition for nutrients. However, there are other variables that may alter this fate, such as the host immune response or the parasite's realized niche. Mixed infection is a topic that has been receiving increasing attention due to the possible impacts on the host. Concerning genotypes of *T. cruzi*, Magalhães and coauthors evaluated the impact of *T. cruzi* DTUs TcIV and TcV on human monocytes (Magalhães et al., 2019). The authors observed that unlike single infections, coinfections resulted in increased expression of IL10 and TNF, which led the authors to conclude that mixed infection has the potential to favor parasite control. Other authors suggest that, in contrast, mixed infections tend to increase pathogenicity. Under natural conditions, the presence of *T. dionisii* and *T. cruzi* TcI, TcII, TcIII, and TcIV has been observed in the cardiac tissue of a child who died of acute orally acquired Chagas disease (Dario et al., 2016). It was not possible in this case to make any prediction on the course of this infection, but the acute course of the disease (3 weeks) followed by death makes one think that in this case, perhaps the effect of the mixed infection would have had the opposite effect (Dario et al., 2016). In fact, the evaluation of the resultant coinfection of distinct *T. cruzi* genotypes is a difficult task due to the numerous variables involved: age, nodule size, the sequence of infections in relation to DTUs, the presence of other parasites, and immunological status, among many others.

By means of a barcoding and next-generation sequencing approach, two cases of coinfections of TcI with TcVI and TcIV were described in non-human primates from Louisiana in the southern USA (Herrera et al., 2019). The same methodological approach was used to test human *T. cruzi* infection, showing that Chagasic patients in Yucatan (Mexico) displayed infections by diverse *T. cruzi* DTUs. The authors detected the presence of TcI, TcII, TcV, and TcVI in single and mixed infections. In fact, 47% of individuals presented infections due to multiple *T. cruzi* DTUs (Villanueva-Lizama et al., 2019).

Still concerning free-ranging wild mammals, small rodents were also able to harbor multiple *T. cruzi* DTUs, as demonstrated by metabarcoding, in addition to conventional PCR and Sanger sequencing (Pronovost et al., 2018). Bats and marsupials have been proposed as bioaccumulators of *Trypanosoma* spp. since they may display infections by several trypanosome species from different clades: *T. cruzi, T. dionisii, T. lainsoni*, and *T. genarii* (Rodrigues et al., 2019).

The use of blood clots for the extraction of *Trypanosoma* spp. DNA and nested PCR using generalist primers followed by Sanger sequencing demonstrated to be a reliable parasitological method that is more accessible than next-generation sequencing, which is still costly, especially for developing countries. Using this protocol, it was possible to access an expressive *Trypanosoma* diversity and to identify trypanosomes not cultivable in axenic medium and/or mixed infections of animals with low parasitemia (Rodrigues et al., 2019).

## A Promising Interactive Tool to Unravel *T. cruzi* Biology

Studying biological phenomena without considering the scenario in which they occur results in the loss of precious and potentially enlightening information. The determination of the spatial distribution of the elements that compose the epidemiological chain of a parasitic disease is of pivotal importance for the determination of trends and risk evaluation. Moreover, it is worth mentioning that the attempts to control a given multihost parasite that displays a huge intraspecific heterogeneity and a complex transmission cycle as expressed by different epidemiological and enzootiological scenarios employing one single measure will be insufficient. The sustainability of successful control of Chagas in multiple current epidemiological scenarios requires multidisciplinary studies. All of the host species select subpopulations of *T. cruzi* in a unique way and present different infection patterns that depend on numerous macro (landscape) and micro (individual peculiarities) variables.

The study of landscape by the classic methodology of mapping by means of discrete typing units and sharp boundaries does not consider transition areas. Nevertheless, environmental and biological phenomena are typically continuous and exhibit a gradual transition from one to another. The fuzzy logic developed by Zadeh in the 1960s is able to solve the strongly non-linear nature of uncertainty and subjectivity inherent in biological data. Furthermore, the spatial analysis by the fuzzy inference method is a cartographic approach that makes it possible to model the spatial distribution of continuous biological phenomena, representing these distribution levels. Modeling by fuzzy logic allows the incorporation of multidisciplinary expert knowledge into the evaluation process. Despite posing as a paradigm break in comparison to the valuation methodologies based on classic logic, fuzzy theory facilitates dialog between the professionals of exact sciences, responsible for computational implementation, and experts from different biological areas because it allows the use of linguistic variables and simplistic logical rules. Such logic has been vastly used in biological modeling due to its peculiar features, especially the capacity to model complex and non-linear problems in a simple way.

Fuzzy logic is a novel approach for Chagas disease risk prediction. This model already demonstrated the possibility of identifying areas with different degrees of risk, thus allowing a continuous and integrated representation of the variables involved in *T. cruzi* transmission in nature. The output data obtained can be used to support decision-making in epidemiological surveillance of Chagas disease and are certainly an example that can be applied to several other parasite infections in distinct areas (Xavier et al., 2016). Fuzzy models are highly promising for evaluating *T. cruzi* (and potentially all other parasite species as well as transmission risk areas). The term “map algebra” was established by Dana Tomlin in the early 1980s (Tomlin, 1990) with the development of the “Map Analysis Package GIS.” Map algebra provides tools to perform spatial analysis operations and is based on matrix algebra, which refers to the algebraic manipulation of matrices (as maps in raster data structures). Spatial analysis by the interpolation method, followed by map algebra, is able to model the spatial distribution of biological phenomena and their distribution and eventual association with other parameters or variables, enhancing the decision power of responsible authorities. Acute Chagas disease outbreaks are increasing in the Amazon basin as a result of oral transmission. This scenario requires a new approach to identify hotspot transmission areas and implement control measures. A geospatial approach using interpolation and map algebra methods to evaluate mammalian fauna was demonstrated to be a reliable strategy to detect hotspot transmission areas in the wild. The construction of maps with mammalian fauna variables, including the infection rates by *T. cruzi*, in dogs and in small wild mammal species, demonstrated that a high prevalence of *T. cruzi* infection in dogs and small wild mammals was associated with a lower richness in mammals. Consequently, it was shown that monitoring *T. cruzi* infection in dogs may be a valuable tool for detecting a lower richness of small wild mammals in the fauna and elucidating the transmission cycle of *T. cruzi* in the wild. The results obtained by visual examination of the maps were validated by statistical analysis (Xavier et al., 2012).

## Concluding Remarks

Here, we briefly described some features that have been landmarks in the course of knowledge building on *Trypanosoma cruzi*. Because it is such a complex parasite, *T. cruzi*, to date, still displays contradictory or even obscure biology. While the main attributes of *T. cruzi* (infectivity and disease in humans, wild and domestic hosts, transmission to humans by domicile adapted triatomines) were revealed in a few years by Carlos Chagas, the *T. cruzi* diversity, pathogenesis, peculiar features of the interaction of *T. cruzi* with its numerous hosts and its multiplication and dispersion strategies still constitute a puzzle. Undoubtedly, the huge technological advance and the consequent stronger analytical power allowed the resolution of many of these issues. However, the current epidemiological profile of Chagas disease, due to the oral route, constitutes a challenge and a scourge, especially for people with less access to adequate sanitary conditions and medical care. One of the possible obstacles to controlling this parasitosis does not depend solely on technology but relies on the conceptual framework of researchers and technicians that is often rigid, poorly resilient and open to the new, disregarding the hyperbolic doubt of René Descartes' (1637) concept about the importance of the need to continually inquire the truth of what is presented as true. This kind of attitude is paralyzing, just as paralyzing as the lack of self-confidence and the hesitation to publish their findings. Science also includes intuition beyond assumptions built on solid foundations. This is exemplified by the pioneering study of an oral outbreak in Nova Teutônia, Santa Catarina State, Southern Brazil. It was a boarding school where several young people became ill and Chagas disease was diagnosed. At the time, all searches for triatomines resulted in vain. The outbreak remained unexplained. One of the authors, Nery-Guimarães, hypothesized that perhaps the ingestion of food that had been contaminated by the urine of *T. cruzi*-infected opossums could be the cause (Nery-Guimarães et al., 1968). It was not far from the truth. In 1984, almost 20 years later, Deane and coauthors described the extracellular cycle of *T. cruzi* in the opossum scent glands of *D. aurita* (Deane et al., 1984a). So also did Carlos Chagas: he made the discoveries that made him famous while in charge of studying malaria in the region where a railway was built. As soon as he was informed by a construction engineer of the blood-sucking insects, he did not hesitate to collect and examine them. He also examined domestic and wild animals as well as humans. Accordingly, he closed the main contours of this new zoonosis.

Technological advancement has resulted in new insights into the parasitism phenomenon and consequently in the reformulation of concepts. An example is the definition of what constitutes a reservoir. Carlos Chagas defined the armadillo as a “depositary,” probably disregarding, as expected at that time, that a host receives and exerts selective pressures on the parasites. The very concepts of reservoir and of what is a parasite have been changing since the days of Carlos Chagas. In this sense, the most didactic and clear definition of what is a reservoir does not refer to the number of species or the ability to cause or not to damage but defines reservoir as one animal species or a set of animal species responsible for maintaining the parasite in the wild. This set of animals will be different for each location and time scale (Jansen et al., 2015; adapted from Ashford, 1997).

Parasitic specificity has also been revisited as a consequence of technological innovation, and the awareness of the importance of including wild animals and their parasites in parasitology studies has increased. Trypanosomatids are no exception: reports of the occurrence of trypanosomatids in unfamiliar hosts are increasing, undermining paradigms of host species and *Trypanosoma* spp. associations. A clear understanding of host-parasite interaction and the mechanisms involved in this adaptation process are important from the academic point of view but also for public health, because in times of profound climate change, emerging diseases result precisely from parasitic spillover to unfamiliar hosts. The acquisition and loss of host species are common events in the parasitism phenomenon and can happen independent of a long-term coevolutionary process. Actually, ecological host fitting is a process of spillover that depends on the preexisting attributes of the parasite (Araujo et al., 2015) and is probably an important driving force for the diversification of generalist parasites such as *T. cruzi*.

The answers to most of the still open questions of *T. cruzi* biology may be found in its ancient hosts, i.e., the wild mammals in nature.

## Author Contributions

AJ and AR wrote the manuscript. SX performed figures. All authors conceived, designed the manuscript, read, and approved the final manuscript.

### Conflict of Interest

The authors declare that the research was conducted in the absence of any commercial or financial relationships that could be construed as a potential conflict of interest.
